# Tuberculosis incidence among migrants according to migrant status: a cohort study, Denmark, 1993 to 2015

**DOI:** 10.2807/1560-7917.ES.2019.24.44.1900238

**Published:** 2019-10-31

**Authors:** Kristina Langholz Kristensen, Troels Lillebaek, Joergen Holm Petersen, Sally Hargreaves, Laura B Nellums, Jon S Friedland, Peter Henrik Andersen, Pernille Ravn, Marie Norredam

**Affiliations:** 1 International Reference Laboratory of Mycobacteriology, Statens Serum Institut, Copenhagen, Denmark; 2 Department of Pulmonary and Infectious Diseases, Nordsjællands Hospital, Hillerød, Denmark; 3 Global Health Section, Department of Public Health, University of Copenhagen, Copenhagen, Denmark; 4 Section of Biostatistics, University of Copenhagen, Copenhagen, Denmark; 5 Institute for Infection & Immunity, St. George’s, University of London, London, United Kingdom; 6 Division of Epidemiology and Public Health, School of Medicine, University of Nottingham, Nottingham, United Kingdom; 7 Department of Infectious Disease Epidemiology and Prevention, Statens Serum Institut, Copenhagen, Denmark; 8 Department of Medicine, Infectious Disease Section, Herlev-Gentofte Hospital, Copenhagen, Denmark; 9 Research Centre for Migration, Ethnicity and Health, University of Copenhagen, Copenhagen, Denmark; 10 Department of Infectious Diseases, Section of Immigrants Medicine, University Hospital Hvidovre, Hvidovre, Denmark

**Keywords:** tuberculosis, migrants, screening, public health, asylum seekers, family-reunified, immigrants, refugees

## Abstract

**Background:**

Migrants account for the majority of tuberculosis (TB) cases in low-incidence countries in western Europe. TB incidence among migrants might be influenced by patterns of migration, but this is not well understood.

**Aim:**

To investigate differences in TB risk across migrant groups according to migrant status and region of origin.

**Methods:**

This prospective cohort study included migrants ≥ 18 years of age who obtained residency in Denmark between 1 January 1993 and 31 December 2015, matched 1:6 to Danish-born individuals. Migrants were grouped according to legal status of residency and region of origin. Incidence rates (IR) and incidence rate ratios (IRR) were estimated by Poisson regression.

**Results:**

The cohort included 142,314 migrants. Migrants had significantly higher TB incidence (IR: 120/100,000 person-years (PY); 95% confidence interval (CI): 115–126) than Danish-born individuals (IR: 4/100,000 PY; 95% CI: 3–4). The IRR was significantly higher in all migrant groups compared with Danish-born (p < 0.01). A particularly higher risk was seen among family-reunified to refugees (IRR: 61.8; 95% CI: 52.7–72.4), quota refugees (IRR: 46.0; 95% CI: 36.6–57.6) and former asylum seekers (IRR: 45.3; 95% CI: 40.2–51.1), whereas lower risk was seen among family-reunified to Danish/Nordic citizens (IRR 15.8; 95% CI: 13.6–18.4) and family-reunified to immigrants (IRR: 16.9; 95% CI: 13.5–21.3).

**Discussion:**

All migrants had higher TB risk compared with the Danish-born population. While screening programmes focus mostly on asylum seekers, other migrant groups with high risk of TB are missed. Awareness of TB risk in all high-risk groups should be strengthened and screening programmes should be optimised.

## Introduction

Improving tuberculosis (TB) care among migrants is considered a key public health priority towards global elimination of TB [1]. Migrants account for the majority of TB in low-incidence countries, defined as those with a notification rate < 10 per 100,000 population per year [1] and comprise over 70% of TB cases in countries like Denmark (incidence: 5/100,000 population/year) and the United Kingdom (UK) (incidence: 9/100,000 population/year) [2,3]. While TB rates in native-born European populations are decreasing, the proportion of cases occurring in migrants is increasing, which may be partly attributed to the recent surge of refugees in some European countries [4]. In this paper, migrants refer to all groups of foreign-born individuals.

Migrants from high-incidence TB countries, defined as notification rate > 20/100,000 population/year [5], are likely to be at increased risk of TB several years following migration because of an increased risk of reactivation of latent TB (LTBI) [6-9]. Some migrants may also be vulnerable to TB because of risk factors associated with migration [10]. Refugees in particular are potentially exposed to poor and dangerous travel conditions, overcrowded housing and poor access to healthcare during flight [11]. After arrival in the host country, potential barriers such as language, lack of knowledge regarding the healthcare system and patient rights, and lack of entitlement can present challenges to accessing the healthcare system [12].

Across Europe, different strategies for TB screening among migrants have been implemented to improve TB detection and treatment, and to reduce the burden of TB in low-incidence countries with programmes focusing on active TB screening before and at arrival [13]. In Denmark, a voluntary medical assessment is provided for asylum seekers when they arrive at the national reception centre. Since 2002, national guidelines have recommended screening for TB among asylum seekers from high-incidence countries, but formalised, systematic screening was not effectuated before 2017 [14]. There is no systematic health or TB screening among quota refugees or family-reunified migrants [15].

A better understanding of the patterns of TB risk among migrants is needed to tailor health services according to needs and to optimise policies for migrants’ contact with healthcare systems. The majority of previous studies have focused on region of origin as a risk factor, but recently there has been a focus on migrant status as such [16]. Migrant status is of interest because exposure to TB risk factors and access to TB screening differs. Only a few studies have assessed TB incidence among subgroups of migrants and how different types of migration affects the risk of TB [9,17].

To investigate differences in risk of TB across migrant groups, we compared TB incidence according to migrant status and region of origin with a Danish-born population in a large-scale cohort study among migrants in Denmark spanning 23 years.

## Methods

### Design and study population

This register-based, prospective cohort study used data obtained from the Danish Immigration Service and from Statistics Denmark. All migrants ≥ 18 years of age who obtained residency in Denmark between 1 January 1993 and 31 December 2015 were included. The age cut-off was used because this is the cut-off between being a child or an adult in Denmark at arrival following the United Nations (UN) Convention on the Rights of the Child [18].

Individuals were included from the date of receiving residency. Date of residency was used for this study because it is an official registered date available for all migrants in Denmark while registered dates of entry are much more uncertain and not available for all migrants. A Danish-born comparison group was identified through Statistics Denmark and matched 1:6 on age and sex based on the first day of the year when the migrant-match was granted residency. Migrants with a diagnosis of TB before the date of residency were excluded for further analysis. The construction of the cohort has previously been described in more detail including the matching procedure and exclusion process because of missing values [19,20].

A TB case was defined as active TB, including both pulmonary and extrapulmonary TB.

Migrants were grouped into five groups based on the legal basis of their residence: (i) former asylum seekers, (ii) quota refugees, (iii) family-reunified to Danish/Nordic citizens, (iv) family-reunified to immigrants, and (v) family-reunified to refugees (Table 1). Migrants were grouped into six groups according to region of origin, with these regions being modified versions of those used by the World Bank Group [21]: (i) Eastern Europe and Central Asia; (ii) Europe, North America and Oceania; (iii) Middle East and North Africa; (iv) Latin America and the Caribbean; (v) South Asia, East Asia and Pacific, hereafter referred to as South-East Asia; and (vi) sub-Saharan Africa (countries represented in the cohort are listed in Supplementary Table S1).

**Table 1:** Migration status terminology used in cohort study of tuberculosis, Denmark, 1993–2015

Term	Definition
**Migrant^a^** [4,40]	Person who is moving or has moved across an international border away from his/her habitual place of residency regardless of his/her legal status, whether the movement was voluntary or involuntary, the cause of movement, and the length of stay outside the country of origin.
-**Refugee** [4,40]	Person who because of a well-founded fear of persecution for reasons of race, religion, nationality, social group or politics, war or violence has been forced to flee his or her country and seeks protection in another country. Status as refugee is obtained when asylum is granted. A refugee can be either a former asylum seeker or a quota refugee.
--Asylum seeker [4,40]	Person who seeks safety from persecution or serious harm in a country other than his/her own country and awaits a decision on the application for refugee status in the receiving country.
--Quota refugee [41] --(Resettlement refugee)	Person who is transferred from one country to another country that has agreed to resettle him/her and ultimately grant him/her permanent settlement as refugee at arrival following an agreement with the United Nations High Commissioner for Refugees or a similar organisation.
**-Immigrant** [41]	Person who has been granted residency through family reunification or employment purpose.
--Family-reunified to Danish/Nordic citizen [41]	Person who has been granted residency and resettlement with a Danish/Nordic citizen spouse or partner who resides in the country of immigration.
--Family-reunified to immigrant [41]	Person who has been granted residency and resettlement with a spouse or partner who has been granted permanent residence in the country of immigration, likely through employment or education.
--Family-reunified to refugee [41]	Person who has been granted residency and resettlement with a spouse or partner who holds refugee status in the country of immigration.
--Labour migrant^b^ [40]	Person who has been granted residency through employment purpose.
-Undocumented migrant^b^ [4]	Person who does not have the necessary documentation to enter or remain legally in a country.

^a^ Migrants refer to the whole group. Subgroups are defined underneath.

^b^ Labour migrants and undocumented migrants are not included in the current study.

### Data generation

All Danish-born individuals are assigned a unique 10-digit central person registration (CPR) number at birth whereas migrants receive a CPR number at date of residency. The CPR number can be used to track individuals through public registries at an individual level. Migrant status and country of origin were obtained from the Danish Immigration Service. Data on age, sex, immigration and emigration were obtained from Statistics Denmark. Data on TB cases were obtained via cross- linkage of CPR numbers through the International Reference Laboratory of Mycobacteriology (IRLM) and the National Surveillance Register (NSR), Department of Infectious Disease Epidemiology and Prevention (DIDE) both at Statens Serum Institut, Copenhagen. In Denmark, all TB diagnostics are centralised at IRLM and nationwide data on culture-verified TB cases were retrieved from here. TB is a notifiable disease by law and the treating physician submits a notification to the NSR, DIDE. We retrieved data on all notified TB cases from the NSR, DIDE to ensure the inclusion of culture-negative cases, i.e. patients with clinical or paraclinical findings suggestive of TB and prompting TB treatment. Since HIV is a known risk factor for TB [22], we retrieved HIV diagnoses from the NSR, DIDE in order to characterise risk-profile of the cohort.

### Data analysis

Descriptive analyses were carried out using chi-squared and t-test analyses. Poisson regression analysis was used to calculate incidence rates (IR) and incidence rate ratios (IRR) of TB with 95% confidence intervals (CI) and using the logarithm of follow-up time as offset. Follow-up time was calculated as time from inclusion until: (i) TB diagnosis, (ii) emigration, (iii) death or (iv) study end whichever came first and was used to quantify the number of years a person was at risk of TB.

First, we calculated IRR for TB adjusted for age and sex, and stratified for migrant status and region of origin using Danish-born as the reference group. We identified interactions between sex and migrant status, and between sex and region of origin. We present data as overall for migrants compared with the Danish-born population. In order to determine the potential influence of migrant status, we calculated IRR for TB among migrants in a model adjusted for age, sex and region of origin, and stratified for migrant status and region of origin using asylum seekers as the reference group. We included age as a time-varying covariate and adjusted for age in 10-year intervals.

All analyses were performed in SAS version 9.4 (SAS Institute, Cary, United States (US)).

We reported our study results according to the STROBE guidelines [23].

### Ethical approval

This study was approved by the Danish Data Protection Agency (Number 2016–41–4576). No further ethical approval is required in Denmark for registry-based research. Data were analysed anonymously via online access to Statistics Denmark’s database.

## Results

In total, 142,314 migrants were included in the cohort (Figure). Of these, 40.9% were family-reunified to Danish/Nordic citizens, 37.9% were former asylum seekers and 10.3% were family-reunified to refugees (Table 2). A total of 854,820 Danish-born individuals made up the control cohort. The distribution of region of origin differed. The Middle East and North Africa were the most common region of origin for family-reunified to refugees (42.9%), former asylum seekers (37.6%) and quota refugees (35.9%) whereas Eastern Europe and Central Asia was the most common for family-reunified to immigrants (55.9%) and South-East Asia for family-reunified to Danish/Nordic citizens (33.1%) (p < 0.01). Among migrants, 56.1% were female and the proportion of females was higher among family-reunified to refugees (80.8%), family-reunified to Danish/Nordic citizens (68.3%) and family-reunified to immigrants (64.2%) in contrast to quota refugees (40.5%) and former asylum seekers (36.4%) (p < 0.01).

**Figure:**
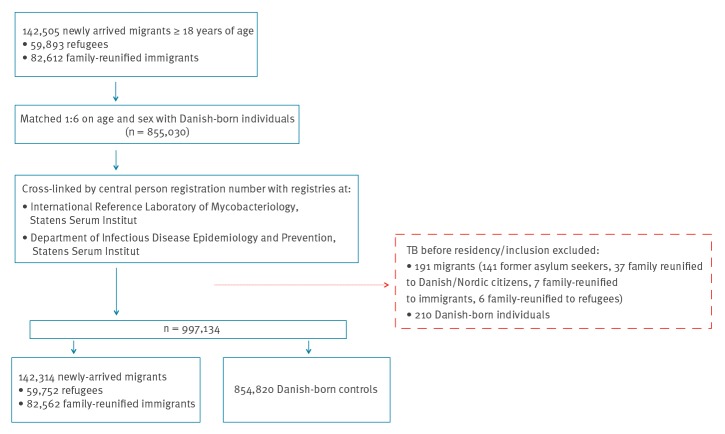
Flow chart of the selection process of the final study cohort, including migrants and Danish-born, Denmark, 1993–2015

**Table 2:** Baseline characteristics of study cohort by migrant status, Denmark, 1993–2015 (n = 997,134)

Characteristics	Danish-born		All migrants		Former asylum seekers		Quota refugees		Family-reunified to Danish/Nordic citizens		Family-reunified to immigrants		Family-reunified to refugees	
	%	n	%	n	%	n	%	n	%	n	%	n	%	n
**Total cohort**	85.7	854,820	14.3	142,314	37.9	53,860	4.1	5,892	40.9	58,135	6.9	9,780	10.3	14,647
**Age at residency (migrants) or cohort inclusion (Danish-born population), years (mean, SD)**	32.7	10.7	32.7	10.7	34.3	11.8	33.1	11.1	31.8	9.2	29.1	10.1	32.4	11.0
**Sex**														
Female	56.1	479,189	56.1	79,784	36.4	19,582	40.5	2,384	68.3	39,706	64.2	6,277	80.8	11,835
Male	43.9	375,631	43.9	62,530	63.4	34,278	59.5	3,508	31.7	18,429	35.8	3,503	19.2	2,812
**Region of origin**														
Eastern Europe and Central Asia	NA	NA	24.4	34,755	33.9	18,263	0.7	41	16.8	9,791	55.9	5,471	8.1	1,189
Europe, North America and Oceania	NA	NA	10.8	15,354	0.4	234	0.0	1	24.2	14,066	6.1	599	3.1	454
Latin America and the Caribbean	NA	NA	3.5	4,955	0.1	44	3.0	177	7.9	4,612	0.9	87	0.2	35
Middle East and North Africa	NA	NA	25.1	35,733	37.6	20,263	35.9	2,119	10.3	5,970	11.2	1,099	42.9	6,282
South-East Asia	NA	NA	22.4	31,911	9.8	5,271	33.8	1,992	33.1	19,238	20.4	1,995	23.3	3,415
Sub-Saharan Africa	NA	NA	13.8	19,606	18.2	9,785	26.5	1,562	7.7	4,458	5.4	529	22.3	3,272
**10 most frequent countries of origin**														
Afghanistan	NA	NA	4.6	6,487	7.9	4,240	6.7	394	0.4	215	0.5	45	10.9	1,593
Bosnia and Herzegovina	NA	NA	10.5	14,918	25.8	13,879	0.0	0.0	0.7	389	0.8	75	3.9	575
Iran	NA	NA	3.7	5,235	4.2	2,249	12.3	723	2.1	1,239	0.8	81	6.4	943
Iraq	NA	NA	8.6	12,166	13.4	7,194	19.8	1,167	1.0	552	0.8	74	21.7	3,179
Philippines	NA	NA	2.6	3,713	0.0	2	0.0	0	6.1	3,521	1.8	172	0.1	18
Russia	NA	NA	2.4	3,359	1.6	876	0.3	20	3.9	2,236	1.0	94	0.9	133
Somalia	NA	NA	6.4	9,144	11.1	5,963	3.8	222	0.3	156	0.5	53	18.9	2,750
Syria	NA	NA	8.4	11,951	18.6	9,989	3.1	181	0.3	200	0.3	33	10.6	1,548
Thailand	NA	NA	5.0	7,133	0.0	1	0.0	0	12.0	6,967	1.4	139	0.3	26
Turkey	NA	NA	6.4	9,087	0.1	58	0.0	2	8.6	4,981	40.3	3,945	0.7	101
**HIV positive**	0.1	401	0.4	540	0.1	74	1.4	83	0.6	333	0.3	25	0.2	25
**Follow-up time, years (mean, SD)**	12.4	7.1	7.3	10.8	11.0	8.0	11.1	6.6	10.0	6.6	13.3	6.8	11.3	6.9
**Events follow-up**														
Tuberculosis cases	0.04	398	1.3	1,841	1.7	923	2.1	125	0.6	372	0.9	92	2.3	329
Death	3.6	30,565	2.5	3,559	4.1	2,227	3.0	176	1.3	738	1.9	182	1.6	236
Emigration	0.0	0	14.3	20,365	10.1	5,541	7.6	449	19.3	11,218	11.7	1,147	14.4	2,110
Population at study end	96.4	823,857	81.9	116,549	84.1	45,269	87.3	5,142	78.8	45,807	85.5	8,359	81.7	11,972

NA: not applicable; SD: standard deviation.

Data are presented as % (n) unless otherwise stated.

We found 1,841 TB cases among migrants and 398 among the Danish-born population. Characteristics are presented in Table 3. The distribution of region of origin differed among groups with most former asylum seekers (71.4%) and family-reunified to refugees (75.4%) being from sub-Saharan Africa, whereas most quota refugees (51.2%), family-reunified to Danish/Nordic citizens (66.1%), and family-reunified to immigrants (44.6%) were from South-East Asia (p < 0.01) (Table 3). Among migrants, 53.3% of TB cases were female (p < 0.01), whereas 39.2% of TB cases among the Danish-born population were female (p < 0.01). Among migrant groups, the proportion of females was higher among family-reunified to refugees (73.2%) and family-reunified to Danish/Nordic citizens (71.5%) in contrast to among former asylum seekers (42.8%) and quota refugees (25.6%) (p < 0.01).

**Table 3:** Baseline characteristics of tuberculosis cases^a^ in study cohort by migrant status, Denmark, 1993–2015 (n = 2,239)

Characteristics	Danish-born		All migrants		Former asylum seekers		Quota refugees		Family-reunified to Danish/Nordic citizens		Family-reunified to immigrants		Family-reunified to refugees	
	%	n	%	n	%	n	%	n	%	n	%	n	%	n
**Age at diagnosis, years (mean, SD)**	42.1	10.8	36.0	12.2	36.9	13.1	35.9	12.2	34.6	9.1	33.9	11.5	35.8	12.6
**Time to diagnosis, years (mean, SD)**	8.8	5.6	4.3	4.1	4.3	4.2	4.3	3.8	4.6	4.1	4.4	4.2	3.9	3.9
**Total TB cases**	100	398	100	1,841	100	923	100	125	100	372	100	92	100	329
**Sex**														
Female	39.2	156	53.3	982	42.8	395	25.6	32	71.5	266	52.2	48	73.2	241
Male	60.8	242	46.7	859	57.2	528	74.4	93	28.5	106	47.8	44	26.8	88
**Region of origin**														
East Europe and Central Asia	NA	NA	10.7	197	14.6	135	0.0	0	7.0	26	34.8	32	1.2	4
Europe, North America and Oceania	NA	NA	0.8	15	0.2	2	0.0	0	2.4	9	2.2	2	0.6	2
Middle East and North Africa	NA	NA	5.5	102	5.5	51	10.4	13	3.5	13	7.6	7	5.5	18
Latin America and the Caribbean	NA	NA	0.3	6	0.1	1	0.0	0	1.3	5	0.0	0	0.0	0
South-East Asia	NA	NA	26.2	483	8.1	75	51.2	64	66.1	246	44.6	41	17.3	57
Sub-Saharan Africa	NA	NA	56.4	1,038	71.4	659	38.4	48	19.6	73	10.9	10	75.4	248
**10 most frequent countries of origin**														
Afghanistan	NA	NA	4.7	86	3.1	57	3.2	4	0.3	1	0.0	0	7.3	24
Bosnia and Herzegovina	NA	NA	5.6	104	11.2	103	0.0	0	0.0	0	1.1	1	0.0	0
Iraq	NA	NA	3.2	58	4.1	38	4.8	6	0.5	2	0.0	0	3.6	12
Myanmar/Burma	NA	NA	2.2	40	0.2	2	25.6	32	0.0	0	0.0	0	1.8	6
Pakistan	NA	NA	3.7	69	0.1	1	0.0	0	12.9	48	18.5	17	0.9	3
Philippines	NA	NA	3.2	59	0.0	0	0.0	0	14.5	54	5.4	5	0.0	0
Somalia	NA	NA	47.1	873	66.9	617	9.6	12	1.3	5	5.4	5	71.1	234
Thailand	NA	NA	4.6	85	0.0	0	0.0	0	21.2	79	6.5	6	0.0	0
Turkey	NA	NA	2.2	40	0.2	2	0.0	0	4.3	16	22.8	21	0.3	1
Vietnam	NA	NA	2.7	51	0.2	2	1.6	2	7.8	29	4.3	4	4.2	14
**HIV positive**	2.0	8	2.3	42	0.4	4	6.4	8	6.2	23	4.3	4	0.9	3
**Disease site^b^**														
Pulmonary tuberculosis	87.4	348	52.4	965	51.6	476	48.8	61	56.5	210	59.8	55	49.5	163
Extrapulmonary tuberculosis	10.3	41	46.3	852	47.8	442	42.4	53	42.2	157	40.2	37	49.5	163

NA: not applicable; SD: standard deviation.

^a^ All cases of tuberculosis: all active tuberculosis cases, including both pulmonary and extrapulmonary tuberculosis.

^b^ Site unknown not listed.

Data are presented as n (%) unless otherwise stated.

Among TB cases, the prevalence of HIV among migrants was 2.3% and among the Danish-born population 2.0% (p = 0.72). The highest prevalence of HIV was seen among quota refugees (6.4%) and family-reunified to Danish/Nordic citizens (6.2%) (p < 0.01).

Overall, the IR of TB among migrants was 120 per 100,000 person-years of follow-up (PY) (95% CI: 115–126) compared with the Danish-born population who had an IR of 4 per 100,000 PY (95% CI: 3–4) (Table 4). The IR was highest among family-reunified to refugees (199/100,000 PY; 95% CI: 179–221), quota refugees (192/100,000 PY; 95% CI: 161–229) and former asylum seekers (156/100,000 PY; 95% CI: 146–167) (p < 0.01). According to region of origin, the IR was highest among migrants from sub-Saharan Africa (575/100,000 PY; 95% CI: 541–611) with especially high IR among migrants from Somalia (849/100,000 PY; 95% CI: 794–907). From Somalia, former asylum seekers (883/100,000 PY; 95% CI: 816–955) and family-reunified to refugees (787/100,000 PY; 95% CI: 693–895) had the highest IR, whereas family-reunified to Danish/Nordic citizens had the lowest IR (575/100,000 PY; 95% CI: 239–1,382).

**Table 4:** Incidence rate and incidence rate ratio of tuberculosis cases^a^ among migrants by migrant status and region of origin, Denmark, 1993–2015 (n = 997,134)

Characteristics	Total (n)	TB cases (n)	IR per 100,000 PY (95% CI)	Crude IRR (95% CI)	Adjusted IRR^b^ (95% CI)
**Migrant status**					
Danish-born population	854,820	398	**4 (3–4)**	1 (ref)	1 (ref)
Migrants	142,314	1,841	**120 (115–126)**	**32.0 (28.7–35.7)**	**32.4 (29.0–36.1)**
Former asylum seekers	53,860	923	**156 (146–167)**	**41.6 (37.0–46.9)**	**45.3 (40.2–51.1)**
Quota refugees	5,892	125	**192 (161–229)**	**51.1 (41.8–62.5)**	**46.0 (36.6–57.6)**
Family-reunified to Danish/Nordic citizen	58,135	372	**64 (58–71)**	**17.1 (14.9–19.7)**	**15.8 (13.6–18.4)**
Family-reunified to immigrant	9,780	92	**71 (57–87)**	**18.8 (15.0–23.6)**	**16.9 (13.5–21.3)**
Family-reunified to refugee	14,647	329	**199 (179–221)**	**53.0 (45.8–61.4)**	**61.8 (52.7–72.4)**
**Region of origin**					
Denmark	854,820	398	**4 (3–4)**	1 (ref)	1 (ref)
Eastern Europe and Central Asia	34,755	197	**40 (35–46)**	**10.6 (8.9–12.6)**	**10.5 (8.8–12.5)**
Europe, North America and Oceania	15,354	15	**9 (6–15)**	**2.5 (1.5–4.1)**	**2.7 (1.6–4.5)**
Latin America and the Caribbean	4,955	6	**14 (6–31)**	**3.7 (1.6–8.2)**	**4.1 (1.8–9.1)**
Middle East and North Africa	35,733	102	**32 (26–38)**	**8.4 (6.8–10.5)**	**8.2 (6.5–10.2)**
South-East Asia	31,911	483	**148 (135–162)**	**39.4 (34.5–45.0)**	**41.5 (36.2–47.6)**
Sub-Saharan Africa	19,606	1,038	**575 (541–611)**	**153.0 (136.3–171.8)**	**151.2 (134.4–170.2)**
**10 most frequent countries of origin among TB cases**					
Denmark	854,820	398	**4 (3–4)**	1 (ref)	1 (ref)
Afghanistan	6,487	86	**119 (96–147)**	**31.8 (25.1–40.1)**	**31.3 (24.7–39.7)**
Bosnia and Herzegovina	14,918	104	**42 (35–51)**	**11.3 (9.1–14.0)**	**11.9 (9.5–14.8)**
Iraq	12,166	58	**33 (26–43)**	**8.9 86.8–11.7)**	**8.9 (6.7–11.9)**
Myanmar/Burma	1,188	40	**418 (307–570)**	**111.4 (80.5–154.2)**	**99.0 (69.5–140.9)**
Pakistan	2,942	69	**200 (158–253)**	**53.3 (41.3–68.8)**	**47.3 (36.5–61.4)**
Philippines	3,713	59	**193 (150–249)**	**51.5 (39.2–67.7)**	**57.1 (35.7–91.3)**
Somalia	9,144	873	**849 (794–907)**	**226.0 (200.7–254.4)**	**222.7 (197.4–251.3)**
Thailand	7,133	85	**125 (101–154)**	**33.2 (26.3–42.0)**	**71.3 (52.6–96.6)**
Turkey	9,087	40	**34 (25–46)**	**9.0 (6.5–12.5)**	**7.5 (5.4–10.5)**
Vietnam	2,183	51	**195 (148–257)**	**52.0 (38.9–69.6)**	**54.8 (39.3–76.2)**

CI: confidence interval; IR: incidence rate; IRR: incidence rate ratio; PY: person-years; ref: reference group for comparison; TB: tuberculosis.

^a^ All cases of tuberculosis: all active tuberculosis cases, including both pulmonary and extrapulmonary tuberculosis.

^b^ Adjusted for age and sex.

Bold indicates p < 0.01.

Compared with the Danish-born population, the adjusted IRR of TB was significantly higher among all migrant groups (p < 0.01). Especially high IRR were seen among family-reunified to refugees (61.8; 95% CI: 52.7–72.4), quota refugees (46.0; 95% CI: 36.6–57.6) and former asylum seekers (45.3; 95% CI: 40.2–51.1). When looking at region of origin, the IRR were significantly higher among all regions of origin (p < 0.01), with especially high IRR among migrants from sub-Saharan Africa (151.2; 95% CI: 134.4–170.2) and South-East Asia (41.5; 95% CI: 36.2–47.6).

Table 5 shows IR and IRR of TB among migrants by migrant status and region of origin. Overall, family-reunified to refugees, quota refugees and former asylum seekers had the highest IR. After adjusting for age, sex and region of origin, the IRR of TB was significantly lower for family-reunified to Danish/Nordic citizens (0.45; 95% CI: 0.39–0.80) and family-reunified to immigrants (0.64; 95% CI: 0.51–0.80) compared with former asylum seekers. There was no difference between quota refugees, family-reunified to refugees and former asylum seekers.

**Table 5:** Incidence rates and incidence rate ratios of tuberculosis cases^a^ among migrants by migrant status and region of origin^b^, Denmark, 1993–2015 (n = 142,314)

Migrant status	Total	Eastern Europe and Central Asia	Middle East and North Africa	South-East Asia	Sub-Saharan Africa
**IR per 100,000 PY (95% CI)**					
Former asylum seeker	**156 (146–167)**	**46 (39–54)**	**35 (26–45)**	**120 (96–151)**	**790 (732–853)**
Quota refugee	**192 (161–229)**	0	**21 (24–71)**	**325 (254–415)**	**368 (278–489)**
Family-reunified to:					
- Danish/Nordics citizens	**64 (58–71)**	**24 (17–36)**	**21 (12–36)**	**136 (120–154)**	**166 (132–209)**
- immigrants	**71 (57–87)**	**40 (28–56)**	**50 (24–106)**	**179 (131–243)**	**191 (103–356)**
- refugees	**199 (179–221)**	**27 (10–73)**	**27 (17–42)**	**137 (106–178)**	**707 (624–801)**
**Adjusted IRR^c^ (95% CI)**					
Former asylum seeker	1 (ref)	1 (ref)	1 (ref)	1 (ref)	1 (ref)
Quota refugee	0.81 (0.67–0.98)	0	1.25 (0.68–2.29)	**2.56 (1.83–3.57)**	**0.45 (0.34–0.61)**
Family-reunified to:					
- Danish/Nordics citizens	**0.45 (0.39–0.51)**	**0.42 (0.28–0.64)**	0.57 (0.31–1.05)	1.20 (0.91–1.55)	**0.22 (0.17–0.28)**
- immigrants	**0.64 (0.51–0.80)**	**0.65 (0.44–0.95)**	1.39 (0.63–3.07)	1.44 (0.98–2.11)	**0.28 (0.15–0.53)**
- refugees	0.90 (0.79–1.03)	0.53 (0.19–1.42)	0.80 (0.46–1.37)	1.17 (0.83–1.67)	0.95 (0.82–1.11)

CI: confidence interval; IR: incidence rate; IRR: incidence rate ratio; PY: person-years; ref: reference group for comparison.

^a^ All cases of tuberculosis: all active tuberculosis cases, including both pulmonary and extrapulmonary tuberculosis.

^b^ The regions of Latin America and the Caribbean, and Europe, North America and Oceania were not included in the analysis because of insufficient power.

^c^ Adjusted for age, sex and region of origin.

Bold indicates p < 0.05.

In the stratified analysis for migrant status and region of origin, the highest IR was seen for migrants from sub-Saharan Africa, particularly among former asylum seekers, family-reunified to refugees and quota refugees (p < 0.01) (Table 5). Among migrants from sub-Saharan Africa, the adjusted IRR was significantly lower for quota refugees (0.45; 95% CI: 0.34–0.61), family-reunified to Danish/Nordic citizens (0.22; 95% CI: 0.17–0.28) and family-reunified to immigrants (0.28; 95% CI: 0.15–0.53) compared with former asylum seekers. There was no difference between former asylum seekers and family-reunified to refugees (0.95; 95% CI: 0.82–1.11).

## Discussion

Overall, all migrants in this study experienced significantly higher TB incidence compared with Danish-born across all migrant statuses and regions of origin. The study showed a difference in TB risk across migrant groups with higher risk among family-reunified to refugees, quota refugees and former asylum seekers, whereas family-reunified to Danish/Nordic citizens and family-reunified to immigrants had lower risks.

Other studies have also found a high risk of TB among migrants [9,17,24-26]. The TB incidence in our migrant population was 120 per 100,000 PY (95% CI: 115–126) over a mean study period of 10.8 years, and was 30 times higher than the incidence among the Danish-born population. This incidence was higher than that of a study among migrants in Norway (80/100,000 PY; 95% CI: 76–84) following migration [26]. One potential reason for the difference could be that the Norwegian cohort included migrants as young as 5 years of age, which could contribute to a lower TB incidence. A study among migrants in the UK found a TB incidence of 147 per 100,000 PY (95% CI: 140–154) following migration [9], which was higher than the TB incidence in our cohort. In the UK study, migrants were predominantly from TB high-risk countries, whereas the migrants in our study were from both low- and high-risk countries (Supplementary Table S1).

Some studies have also included the role of migration status as TB risk factor. In a study from Canada, being a refugee or a family-reunified immigrant was a strong predictor of TB following migration compared with being a business or labour immigrant [17]. Asylum seekers and quota refugees were presented as one refugee group. The study from the UK [9] also reported that family-reunified immigrants experienced the highest TB incidence (320/100,000 PY; 95% CI: 244–419) among migrants, which was otherwise comprised of resettlements and dependents, students and working immigrants. This TB incidence was markedly higher than the TB incidence among our family-reunified immigrants (91/100,000 PY; 95% CI: 85–97), but the demographics of family-reunified immigrants in the UK were not specified in more detail so we do not know whether the two populations were comparable.

The higher proportion of females among migrant TB cases than the Danish-born population is likely to reflect an overall difference in the distribution of sex in the migrant population with more females than males resettling in Denmark. The calculated TB risk was slightly lower among female than male migrants (IRR: 0.82; 95% CI: 0.75–0.90, data not shown), which is consistent with what was found in western Sweden [27]. When looking at migrant status, the proportions differed. Male and female migrants differ in their patterns of migration which reflects in the sex distribution. Most male migrants arrive as asylum seekers or quota refugees, whereas many female migrants arrive as either family-reunified to refugees or family-reunified to Danish/Nordic citizens. Among the Danish-born population, TB cases were most often in males, consistent with the general pattern in Denmark, where TB among the Danish-born population is associated with risk factors such as male sex and social marginalisation [28].

High rates of TB according to region of origin are well known from TB surveillance data in low incidence TB countries in western Europe. Migrants from TB endemic countries, particularly from countries in sub-Saharan Africa and South-East Asia, have consistently been shown to have elevated TB rates following migration [9,22,29]. In our study, nearly half of migrant TB cases were from Somalia and thereby contributed largely to the risk of TB. Another important aspect was that TB cases among former asylum seekers and family-reunified to refugees were mainly from sub-Saharan Africa, especially Somalia (68.0%), largely driving the risk of TB in these migrant groups. Somalia is a high-incidence country (IR: 266/100,000 PY [3]) and Somali migrants constitute a long-term, high-risk group in Scandinavian countries [27,30,31]. However, even though Somalia is a high-incidence country, the incidence in our cohort among asylum seekers from Somalia was much higher than the reported incidence in Somalia. In contrast, family-reunified to Danish/Nordic citizens from Somalia had lower incidence. This may possibly reflect risk factors among asylum seekers related to migration such as history of imprisonment, overcrowding and precarious living conditions, compared to family-reunified to Danish/Nordic citizens who typically travel under less strenuous means and arrive directly from their country of origin. This association with migrant status among refugees from Africa has also been observed elsewhere. Asylum seekers from western Africa arriving in Italy had a higher yield of TB screening at arrival than the incidence in their country of origin [32]. Although, one important notion is, that reported TB notifications from African countries is likely to be underreported.

Migrants accounted for a larger proportion of HIV than the Danish-born population, consistent with epidemiological HIV patterns in western Europe [33]. Quota refugees and family-reunified to Danish/Nordic citizens comprised the largest groups of HIV positive migrants and the majority came from countries with on-going HIV epidemics, such as Congo, Kenya and Thailand [34]. Most migrants are infected abroad and unaware of their HIV status, and seeking treatment is usually not the reason for migrating [33]. Interestingly, a previous study found that family-reunified to Danish-Nordic citizens were later presenters of HIV than quota refugees [35]. One potential reason may be that quota refugees are offered voluntary HIV testing by the International Organisation for Migration before arrival in Denmark and a potential HIV diagnosis is followed up after arrival. Family-reunified immigrants are not offered any health assessment upon arrival and the late presentation may be a reflection of differences in introduction to the Danish healthcare system, inadequate screening practises or different health-seeking behaviour [35].

TB screening has been implemented in most European countries [13]. Studies show a high risk of TB among migrants upon arrival and that patterns of migration are indicators of the risk of TB [29,36]. One review found a pooled prevalence of TB of 283 per 100,000 screened (95% CI: 216–349) among migrants at arrival [29]. When stratified, the yield among refugees was 577 per 100,000 screened (95% CI: 206–949), among asylum seekers it was 267 per 100,000 screened (95% CI: 194–341) and among other immigrants it was 225 per 100,000 screened (95% CI: 129–322). The other immigrant group were pooled from studies including students, family-reunified immigrants, labour immigrants etc. and therefore did not allow any conclusion solely regarding family-reunified immigrants. Another review reported a pooled TB prevalence of 300 per 100,000 screened (95% CI: 224–396) among asylum seekers at arrival [36]. The differences in TB at arrival may reflect a risk of TB related to migrant status among others, and support our results of differences in TB among migrants following resettlement.

Our study underlines that family-reunified to refugees may be a high-risk group of TB compared with other family-reunified immigrants and should be prioritised in arrival screening. Asylum seekers and quota refugees leave their home under critical events and may face overcrowded camps or prisons, poor nutrition, stress and barriers to healthcare favouring both transmission and reactivation of TB [10]. Family-reunified to refugees migrate under different legal terms than asylum seekers and quota refugees, but given that many migrate under critical events and face refugee camps etc., they might still be a highly vulnerable group compared with other family-reunified migrants. This means that even though they are legally different, the migration pattern for family-reunified to refugees is similar to that of asylum seekers. In contrast, family-reunified to Danish/Nordic citizens and family-reunified to immigrants are more likely to travel under less strenuous means and in addition, arrive within a stronger support system.

Strengthening TB screening programmes upon arrival for high-risk groups might reduce the high rates of TB following migration. If migrants are not all equally introduced to the healthcare system or systematically offered TB screening this may contribute to inequalities among migrants [15]. Among migrants, relevant TB screening is accepted and the uptake is high [37]. Ensuring TB screening is offered to migrants most at risk, i.e. family-reunified to refugees, quota refugees and asylum seekers, would increase equity in relation to host populations in terms of TB prevention and access to healthcare [38].

### Strengths and limitations

Major strengths of this study were the cohort size and the duration of follow-up time. Also, because of the unique possibilities of the Danish CPR number, we could identify and follow a large cohort of migrants on an individual level on a nationwide basis. Further to that, TB data were retrieved from IRLM and NSR, DIDE, which are believed to cover all TB cases diagnosed in Denmark. We therefore believe our data reflect the high TB incidence among migrants after settling in a low-incidence TB country and the differences across groups.

However, there are some limitations to consider. First, we only included migrants ≥ 18 years of age so we cannot make any inference regarding children. At arrival in Denmark, adults and children were received differently at arrival in terms of health assessment and provision of social support during the study period, so only adults were included to minimise potential bias. Second, undocumented migrants were not represented in the study cohort and these individuals may be at high TB risk and experience significant barriers to healthcare. Third, some migrants may have had TB before arrival and before receiving residency. Because valid information on date of arrival in Denmark was not available for all migrants, we included participants based on date of residency, which is when a CPR number that allows for the exact follow-up through registries is assigned. A consequence of this approach could be an underestimation of TB incidence in our migrant population, particularly among former asylum seekers. This is because asylum seekers apply for asylum at arrival in Denmark and there is consequently a delay between arrival and receiving residency. The delay differs during the study period, but is described as between 6 and 12 months in general [39]. Regarding quota refugees and family-reunited immigrants, they hold a residence permit when they arrive in Denmark and there is therefore usually no delay between arrival in Denmark and residency. However, because of the register-based design of this study, we do not know the time span between arrival in Europe and residency in Denmark. Forth, we compare our results to studies from other countries, but the migrant population in Denmark may look different from migrant populations in other European countries because of differences in policies, traditional migration routes, etc.

### Conclusion

All migrants had increased TB risk compared with the Danish-born population. The risk was particularly high for migrants from sub-Saharan Africa, family-reunified to refugees, quota refugees and former asylum seekers. Screening programmes mostly focus on asylum seekers, but other groups at high risk of TB are missed. Thus, awareness of TB in all high-risk groups should be strengthened and TB screening programmes should be optimised. This study suggests a need for a more comprehensive approach, possibly one that incorporates TB screening into a general health evaluation to improve migrant health.

## Supplementary Data

80000 Supplementary Material
