# Supplementary material for: Tuberculosis incidence among migrants according to migrant status: a cohort study, Denmark, 1993 to 2015
Source: Euro Surveill. 2019 Oct 31;24(44):1900238. doi: 10.2807/1560-7917.ES.2019.24.44.1900238 (PMC6836680; doi:10.2807/1560-7917.ES.2019.24.44.1900238)
Supplement: Supplementary Material [file 1900238_KRISTENSEN_SupplementaryMaterial.pdf]

Title: Supplementary Material

Disclaimer: This supplementary material is hosted by *Eurosurveillance* as supporting information alongside the article Tuberculosis incidence among migrants according to migrant status: a cohort study, Denmark, 1993 to 2015, on behalf of the authors, who remain responsible for the accuracy and appropriateness of the content. The same standards for ethics, copyright, attributions and permissions as for the article apply. Supplements are not edited by *Eurosurveillance* and the journal is not responsible for the maintenance of any links or email addresses provided therein.

SUPPLEMENTARY TABLE S1 Region of origin[a] categorized by country of origin[b]

Region of origin	Countries
Eastern Europe and Central Asia	Albania, Belarus, Bosnia and Herzegovina, Cyprus, former Yugoslav Republic, Kazakhstan, Kosovo, Kyrgyzstan, Moldova, Republic of North Macedonia, Russia, Serbia, Tajikistan, Turkmenistan, Turkey, Uzbekistan
Europe, North America and Oceania	Australia, Austria, Belgium, Bulgaria, Croatia, Canada, Czechia, Estonia, Germany, Hungary, Ireland, Italy, Latvia, Lithuania, Malta, Netherlands, New Zealand, Poland, Portugal, Romania, Slovakia, Slovenia, Spain, Switzerland, Ukraine, United Kingdom
Latin America and Caribbean	Argentina, Bahamas, Belize, Brazil, Chile, Colombia, Costa Rica, Cuba, Dominican Republic, Ecuador, El Salvador, Guatemala, Guyana, Haiti, Honduras, Paraguay, Peru, Panama, Saint Lucia, Saint Vincent and the Grenadines, Trinidad and Tobago, Uruguay, Venezuela
Middle East and North Africa	Algeria, Armenia, Azerbaijan, Bahrain, Djibouti, Egypt, Georgia, Iran, Iraq, Israel, Jordan, Kuwait, Lebanon, Libya, Morocco, Oman, Saudi Arabia, Syria, Tunisia, United Arab Emirates, Yemen
South-East Asia	Afghanistan, Bangladesh, Bhutan, Cambodia, China, Fiji, Hong Kong, India, Indonesia, Japan, Kiribati, Laos, Macao, Malaysia, Maldives, Mongolia, Myanmar/Burma, Nepal, North Korea, Pakistan, Papa New Guinea, Philippines, Samoa, Singapore, Solomon Islands, South Korea, Sri Lanka, Taiwan, Thailand, Tonga, Vietnam
Sub-Saharan Africa	Angola, Benin, Botswana, Burkina Faso, Burundi, Cameroon, Cape Verde, Central African Republic, Chad, Cote D'Ivoire, Democratic Republic of the Congo, Eritrea, Ethiopia, Gabon, Ghana, Guinea, Guinea Bissau, Kenya, Lesotho, Liberia, Madagascar, Malawi, Mali, Mauritania, Mauritius, Mozambique, Namibia, Niger, Nigeria, Rwanda, Senegal, Seychelles, Sierra Leone, Somalia, South Africa, South Sudan, Sudan, Eswatini, Tanzania, The Gambia, Togo, Uganda, Zambia, Zimbabwe

[a] Modified from the World Bank Group
[b] Only countries represented in the study cohort are listed
